# Reproducibility of right-to-left shunt quantification using transthoracic contrast echocardiography in hereditary haemorrhagic telangiectasia

**DOI:** 10.1007/s12471-018-1094-4

**Published:** 2018-03-01

**Authors:** V. M. M. Vorselaars, S. Velthuis, M. P. Huitema, A. E. Hosman, C. J. J. Westermann, R. J. Snijder, J. J. Mager, M. C. Post

**Affiliations:** 1 Department of Cardiology, St. Antonius Hospital, Nieuwegein, The Netherlands; 2 Department of Pulmonology, St. Antonius Hospital, Nieuwegein, The Netherlands

**Keywords:** Hereditary haemorrhagic telangiectasia, Pulmonary right-to-left shunt, Transthoracic contrast echocardiography, Pulmonary arteriovenous malformation

## Abstract

**Aim:**

Transthoracic contrast echocardiography (TTCE) is recommended for screening of pulmonary arteriovenous malformations (PAVMs) in hereditary haemorrhagic telangiectasia. Shunt quantification is used to find treatable PAVMs. So far, there has been no study investigating the reproducibility of this diagnostic test. Therefore, this study aimed to describe inter-observer and inter-injection variability of TTCE.

**Methods:**

We conducted a prospective single centre study. We included all consecutive persons screened for presence of PAVMs in association with hereditary haemorrhagic telangiectasia in 2015. The videos of two contrast injections per patient were divided and reviewed by two cardiologists blinded for patient data. Pulmonary right-to-left shunts were graded using a three-grade scale. Inter-observer and inter-injection agreement was calculated with κ statistics for the presence and grade of pulmonary right-to-left shunts.

**Results:**

We included 107 persons (accounting for 214 injections) (49.5% male, mean age 45.0 ± 16.6 years). A pulmonary right-to-left shunt was present in 136 (63.6%) and 131 (61.2%) injections for observer 1 and 2, respectively. Inter-injection agreement for the presence of pulmonary right-to-left shunts was 0.96 (95% confidence interval (CI) 0.9–1.0) and 0.98 (95% CI 0.94–1.00) for observer 1 and 2, respectively. Inter-injection agreement for pulmonary right-to-left shunt grade was 0.96 (95% CI 0.93–0.99) and 0.95 (95% CI 0.92–0.98) respectively. There was disagreement in right-to-left shunt grade between the contrast injections in 11 patients (10.3%). Inter-observer variability for presence and grade of the pulmonary right-to-left shunt was 0.95 (95% CI 0.91–0.99) and 0.97 (95% CI 0.95–0.99) respectively.

**Conclusion:**

TTCE has an excellent inter-injection and inter-observer agreement for both the presence and grade of pulmonary right-to-left shunts.

**Electronic supplementary material:**

The online version of this article (10.1007/s12471-018-1094-4) contains supplementary material, which is available to authorized users.

## Introduction

Transthoracic contrast echocardiography (TTCE) is used to screen for the presence of pulmonary arteriovenous malformations (PAVMs). Over 90% of PAVMs are associated with hereditary haemorrhagic telangiectasia, an inheritable disease characterised by abnormal artery-to-vein connections in the brain, liver or lungs [1, 2]. Most cases are caused by mutations in the *ENG* and *ACVRL1* gene, leading to hereditary haemorrhagic telangiectasia type 1 and type 2 respectively. A rarer mutation is located on the *SMAD4* gene. PAVMs are frequently described in all subgroups but more prevalent in hereditary haemorrhagic telangiectasia type 1. In up to 21% of patients PAVMs are associated with severe neurologic complications, such as brain abscesses and ischaemic stroke [3]. Furthermore, PAVMs can result in hypoxaemia, haemoptysis and migraine. Most patients, however, remain asymptomatic before the development of complications, making screening for PAVMs extremely important in all patients with or suspected of hereditary haemorrhagic telangiectasia [1, 4–7].

TTCE represents a functional measurement in which PAVMs are visualised as a pulmonary right-to-left shunt. Pulmonary right-to-left shunt grade on TTCE is a good predictor for the presence of treatable PAVMs on chest computed tomography (CT) [8]. Importantly, only moderate to large right-to-left shunts seem to have clinical implications [3, 8, 9]. The international hereditary haemorrhagic telangiectasia guidelines recommend TTCE as first-choice screenings test for PAVMs. However, the reliability of TTCE is dependent on the reproducibility in the individual patient. Although the inter-observer variability has been described in a few studies, there has been no research evaluating the reproducibility of TTCE. Therefore, we aimed to evaluate both the inter-injection and inter-observer variability of TTCE in this prospective single-centre study.

## Material and methods

### Study population

We included all consecutive persons screened for the presence of hereditary haemorrhagic telangiectasia and all consecutive hereditary haemorrhagic telangiectasia patients visiting the outpatient clinic for a regular 5‑year follow-up at the Dutch hereditary haemorrhagic telangiectasia centre between February and November 2015. The clinical diagnosis was established according to the Curaçao criteria, which consist of spontaneous and recurrent epistaxis, telangiectasia at characteristic sites, visceral arteriovenous malformations, and a first-degree relative with hereditary haemorrhagic telangiectasia [1]. Genetic testing was offered to all included patients. A definite diagnosis of hereditary haemorrhagic telangiectasia was established in case of three or more Curaçao criteria, or when genetic testing identified the hereditary haemorrhagic telangiectasia causing gene mutation (e.g. *ENG, ACVRL1* or *SMAD4*).

Patients were excluded if TTCE was not complete (e.g. due to intravenous line failure or image storage problems). The study was approved by the local ethics committee (R&D/Z14.059).

### Transthoracic contrast echocardiography

All TTCEs were performed according to the local clinical protocol. TTCEs were conducted on a Philips IE33 ultrasound instrument (S5-1 transducer; Philips Medical Systems, Best, the Netherlands) or a General Electronic Vivid S6 ultrasound instrument (3S transducer; General Electronic Healthcare, Wauwatosa, The United States).

An intravenous line was inserted in the right antecubital vein, 1 ml blood was drawn and 8 ml physiological saline solution and 1 ml air was added. A second syringe was connected by a bi-directional Luer Lock system. In total, 10 ml agitated saline (containing microbubbles) was created by reverse flushing between the connected syringes [3, 8–10]. The patient was positioned in the left lateral position and 5 ml of agitated saline was injected at rest while projecting the four-chamber apical view. After all microbubbles dissolved, this procedure was repeated.

All TTCE videos were blinded for patient data, numbered and stored in a database. The observers viewed all individual contrast injections in random order to minimise the possibility of bias. Two independent cardiologists (observer 1: VV and observer 2: SV) quantified each shunt.

**Fig. 1:**
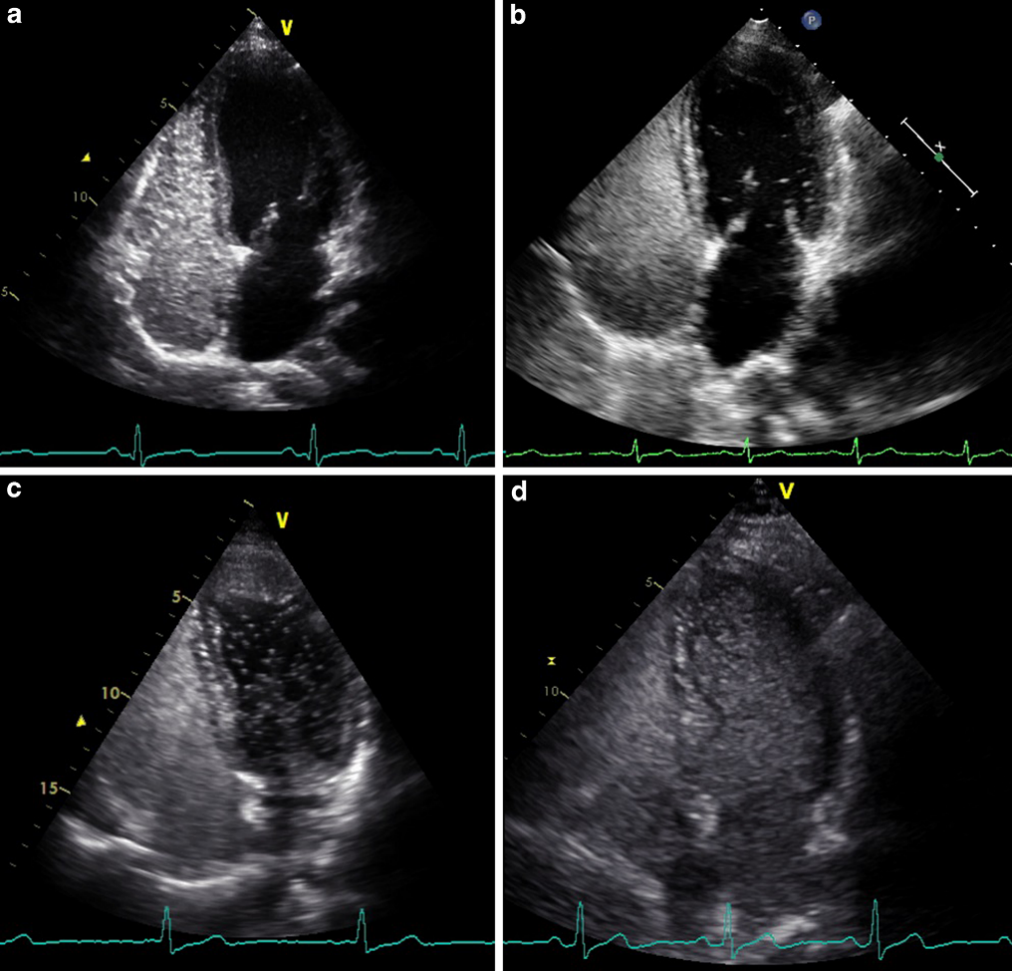
Pulmonary right-to-left shunt (RLS) on transthoracic contrast echocardiogram. **a** No pulmonary RLS. **b** Pulmonary RLS grade 1. **c** Pulmonary RLS grade 2. **d** Pulmonary RLS grade 3

All right-to-left shunts that clearly originated out of the pulmonary vein were classified as pulmonary right-to-left shunts, and all right-to-left shunts appearing through the septum as cardiac right-to-left shunts. If shunt origin was not visible, a delay of four cardiac cycles was used to distinguish between a pulmonary and cardiac shunt. We considered the TTCE positive for a pulmonary right-to-left shunt if microbubbles appeared in the left atrium after four or more cardiac cycles. The pulmonary right-to-left shunt was graded based on the maximum number of microbubbles present in the left ventricle in one still frame. Right-to-left shunt was graded as 1, 2 or 3 corresponding with 1–29, 30–100 and over 100 microbubbles respectively (Fig. 1 and video 1–3) [3, 8, 10–12].

We reviewed the technical quality of the studies and described image quality as good, sufficient or insufficient. Quantity of contrast in the right ventricle was described as sufficient when opacification of the right ventricle was densely filled (with endocardial lining) [13].

### Chest computer tomography

We performed a chest CT examination in all patients with a pulmonary right-to-left shunt grade ≥2 in at least one of the injections [3, 14] and used a ≥16-detector CT scanner (Philips Medical Systems, Best, the Netherlands) with a high-resolution algorithm and slice thickness of 1 mm. All CT images were evaluated by an interventional radiologist and a pulmonologist who were blinded to the TTCE results.

### Statistical analysis

We used descriptive statistics to describe patient characteristics. Continuous variables were reported as mean ± standard deviation. Proportions were given by numbers and corresponding percentages. We measured inter-injection agreement for the presence and grade of a pulmonary right-to-left shunt between the two contrast injections at rest in one patient. Inter-observer agreement was measured for the presence and grade of pulmonary right-to-left shunts between observer 1 and observer 2. We used Cohen’s unweighted kappa coefficient (with 95% confidence intervals (CI)) for nominal characteristics and Cohen’s weighted kappa coefficient (with 95% CI) for ordinal characteristics. Level of agreement was described according to Landis and Koch [15]. Odds ratio (OR) with 95% CI was calculated by performing a binominal logistic regression analyses to describe predictors for inter-injection differences. For the statistics we used a statistical software package (SPSS, version 22; SPSS Inc., Chicago and R (www.r-project.org, version 3.1.2)).

## Results

Between February and November 2015, we used TTCE in 110 patients. After excluding 3 patients (image storage problem *N* = 2, intravenous line failure *N* = 1), 107 patients (49.5% male, mean age 45.0 ± 16.6 years) were included for further analysis (Tab. 1). Image quality of TTCE was good, sufficient and insufficient in 97 (90.7%), 9 (8.4%) and 1 (0.9%) patients/patient respectively. Quantity of contrast opacification of the right ventricle was sufficient in 197 contrast injections (92.1%). A pulmonary right-to-left shunt was present in 136 (63.6%) and 131 (61.2%) injections for observer 1 and 2 respectively. A cardiac right-to-left shunt at rest was present in 3 patients (2.8%) for both observers.

**Table 1:** Baseline characteristics

Number	107
Age	45.0 ± 16.6
Male	53 (49.5%)
*Time of TTCE*	
Screening for HHT^a^	57 (53.3%)
Follow-up of pulmonary RLS^b^	50 (46.7%)
Definite HHT^*c*^	77 (72.0%)
*HHT type*	
– Type 1	32 (29.9%)
– Type 2	36 (33.6%)
– SMAD4	6 (5.6%)
– Unknown	3 (2.8%)

Data are presented as number (%) or mean ± standard deviation

*HHT* hereditary haemorrhagic telangiectasia, *RLS* right-to-left shunt, *SMAD4* SMAD family member 4, *TTCE* transthoracic contrast echocardiography

^a^TTCE made to screen for pulmonary RLS

^b^TTCE made at regular 5 year follow-up

^c^Based on genetic testing or clinical criteria [1]

### Inter-injection agreement

Inter-injection agreement (Tab. 2 and 3) for the presence of pulmonary right-to-left shunt was κ coefficient 0.96; 95% CI 0.90–1.00 (observer 1) and κ coefficient 0.98; 95% CI 0.94–1.00 (observer 2). Inter-injection agreement for pulmonary right-to-left shunt grade was 0.96; 95% CI 0.93–0.99 (observer 1) and κ coefficient 0.95; 95% 0.92–0.98 (observer 2). Observer 1 and observer 2 disagreed about right-to-left shunt grade between first and second contrast injection in 8 and 9 patients respectively. This included 11 patients (10.3%) in total, technical quality of the studies showed good image quality in 8 of these patients (72.7%) and difference in contrast opacification of the right ventricle in 6 patients (54.4%). Disagreement between right-to-left shunt grade 1 and 2 occurred in 3 and 5 patients respectively (5 patients (4.7%) in total). CT scans were taken of all 5 patients and showed a very small PAVM in 1 patient with no possibility for percutaneous treatment. Difference between two injections was never more than one grade. Quantity of contrast in the right ventricle was a predictor for inter-injection disagreement (OR 6.6; 95% CI 1.5–29.8, *p* = 0.01).

**Table 2:** Overview agreement

	Presence of pulmonary RLS	Pulmonary RLS grade
*Inter-injection agreement* [1]		
Kappa	0.96 (0.90–1.00)	0.96 (0.93–0.99)
Absolute agreement	105 (98.1%)	99 (92.5%)
*Inter-injection agreement* [2]		
Kappa	0.98 (0.94–1.00)	0.95 (0.92–0.98)
Absolute agreement	106 (99.1%)	98 (91.6%)
*Inter-observer agreement*		
Kappa	0.95 (0.91–0.99)	0.97 (0.95–0.99)
Absolute agreement	209 (97.7%)	203 (94.9%)

Absolute agreement is described as number with percentage, other agreements are described as kappa with 95% confidence interval. (1): observer 1; (2): observer 2

*RLS* right-to-left shunt

**Table 3:** Inter-injection and inter-observer agreement

**Inter-injection agreement, observer 1**						
		**Injection 1**				
		No RLS	RLS grade 1	RLS grade 2	RLS grade 3	Total
**Injection 2**	No RLS	38	1	0	0	39
	RLS grade 1	1	39	0	0	40
	RLS grade 2	0	3	14	0	17
	RLS grade 3	0	0	3	8	11
	Total	39	43	17	8	107
**Inter-injection agreement, observer 2**						
		**Injection 1**				
		No RLS	RLS grade 1	RLS grade 2	RLS grade 3	Total
**Injection 2**	No RLS	41	0	0	0	41
	RLS grade 1	1	38	0	0	39
	RLS grade 2	0	5	12	1	18
	RLS grade 3	0	0	2	7	9
	Total	42	43	14	8	107
**Inter-observer agreement**						
		**Observer 1**				
		No RLS	RLS grade 1	RLS grade 2	RLS grade 3	Total
**Observer 2**	No RLS	78	5	0	0	83
	RLS grade 1	0	78	4	0	82
	RLS grade 2	0	0	30	2	32
	RLS grade 3	0	0	0	17	17
	Total	78	83	34	19	214

*RLS* right-to-left shunt

### Inter-observer agreement

Inter-observer agreement (Tab. 2 and 3) for pulmonary right-to-left shunt presence and grade was κ coefficient 0.95; 95% CI 0.91–0.99 and κ coefficient 0.97; 95% CI 0.95–0.99 respectively.

### Complications

During placement of an intravenous line, one patient (0.9%) experienced vagal symptoms and an irregular heart rate. Rhythm on TTCE showed atrial fibrillation, which was confirmed with a 12-lead electrocardiogram. During TTCE there were no major complications. One patient (0.9%), with a right-to-left shunt grade 2, reported dizziness in the first hour after TTCE with spontaneous full recovery.

## Discussion

This is the first study that describes the reproducibility of the detection and quantification of pulmonary right-to-left shunt on TTCE in patients screened for the presence of PAVM in association with hereditary haemorrhagic telangiectasia. We found a high level of agreement for the detection and grading of a pulmonary right-to-left shunt in an individual patient (κ coefficient 0.95–0.98).

The use of TTCE to detect intra-pulmonary right-to-left shunts was first described in 1976 [16]. The technique for TTCE is based on the permeability of the pulmonary capillary network and the difference in density between gas-contained microbubbles and the surrounding blood [17, 18]. The capillary network normally measures 8 to 10 µm in diameter, and therefore the injected microbubbles with a mean diameter of 27 μm will be trapped in the pulmonary circulation. If PAVMs are present the filtering capacity of the capillary network will be diminished, and microbubbles will pass the pulmonary filter and appear in the left side of the heart [9].

Clinical implications of the use of TTCE in hereditary haemorrhagic telangiectasia have emerged in the last few years. Gazzaniga et al. described an association between pulmonary right-to-left shunt grade and the occurrence of complications (haemoptysis, cerebral abscesses and stroke) [11]. This was confirmed in a large multicentre study that included over 1,000 patients and described pulmonary right-to-left shunt grade 2 and 3 as independent predictors for the prevalence of a cerebral ischaemic event or brain abscess (OR 4.78; *p* = 0.03 and OR 10.4, *p* = 0.002) [3]. The pulmonary right-to-left shunt grade also predicts the size of PAVMs on CT and the subsequent feasibility of embolisation [8]. This suggests that only moderate and large right-to-left shunts have clinical implications. Therefore, CT can be safely withheld in patients with a grade 1 pulmonary right-to-left shunt reducing radiation exposure for many hereditary haemorrhagic telangiectasia patients [8, 19]. Within 5 years, no treatable PAVMs develop in patients without a pulmonary right-to-left shunt at screening. However, increase in pulmonary right-to-left shunts occurs in approximately 18%, leading to the need for embolisation in 12% of patients with initially non-treatable PAVMs at screening [20].

Although the above data already described the importance of TTCE, reliability of TTCE is based on the inter-observer variability and reproducibility in the individual patient. Many studies already described a high inter-observer agreement (κ coefficient 0.85–0.94) for pulmonary right-to-left shunt detection and, therefore, our results are in line with the previous [11, 12, 21].

This is the first study describing the inter-injection agreement for pulmonary right-to-left shunt quantification since none of the previous studies consecutively performed multiple injections in one single patient. Although not completely comparable, repeated injections for the diagnosis of a cardiac right-to-left shunt have shown to increase the detection of right-to-left shunts due to a high number of false negative injections [13, 22]. Differences in quantification of cardiac right-to-left shunts were mainly based on the technique of provocation, insufficient contrast in the right ventricle and poor image quality. The inter-observer and intra-observer agreements for the detection of a cardiac right-to-left shunt seem much lower (0.77 and 0.82 respectively) in comparison with those found for pulmonary right-to-left shunts [23]. This may be explained by the different mechanisms of both shunts. In contrast to the cardiac right-to-left shunt, the pulmonary right-to-left shunt represents a persistent shunt and therefore no provocation is required to visualise the right-to-left shunt. However, our study confirms the need for sufficient contrast opacification of the right ventricle to obtain a reliable result, as it is shown to be a predictor for inter-injection disagreement (OR 6.6; 95% CI 1.5–29.8, *p* = 0.01). This confirms the utmost importance of adequately producing and injecting the agitated saline. Obtaining a good acoustic window is essential for reliable right-to-left shunt quantification [24]. Performance of a second contrast injection should therefore be recommended when any doubt on right-to-left shunt grade exists. TTCE may also be used to diagnose a pulmonary right-to-left shunt related to hepatopulmonary syndrome. Although there are pathophysiological differences, the results of our study may be translated to these patients.

Although this study showed an almost perfect level of inter-injection agreement, it should be emphasised that there was disagreement between the first and second contrast injection in 11 patients (10.3%). This might have clinical implications when treatable PAVMs are missed. Since CT is withheld in patients with a pulmonary right-to-left shunt <2, quantification of a right-to-left shunt grade 1 instead of grade 2 might have consequences. In this study, this occurred in 5 patients (4.7%). CT did not demonstrate treatable PAVMs in any of these patients. This reinforces our previously described recommendation to exclude right-to-left shunts grade 1 from further CT analysis.

Some clinicians have concerns regarding the safety of the contrast injection, especially in patients with a right-to-left shunt. However, the microbubbles in the injected agitated saline are very small and implode easily. Multiple recent studies in patients with and without hereditary haemorrhagic telangiectasia confirm the safety of TTCE and describe only minor and self-resolving side effects such as dizziness and migraine in 0–2% [11, 19, 25, 26]. In line with previous studies, our study showed no severe side effects.

At this moment TTCE is the cornerstone in the diagnostic evaluation of pulmonary right-to-left shunts in the context of hereditary haemorrhagic telangiectasia. However, chest CT remains the gold standard for pre- and post-embolisation evaluation of PAVMs because it provides essential information on the PAVM anatomy (localisation, complexity, size of feeding arteries and aneurysmal sac). Magnetic resonance imaging is also used for PAVM detection. A big advantage compared with chest CT is the avoidance of radiation while the anatomy of the PAVMs can still be demonstrated. Although some studies show promising results, large comparative prospective studies are lacking and the lower spatial resolution (compared with CT) is currently a major limiting factor [27–29].

### Limitations

First, this is a single-centre study performed in a tertiary referral hospital that is highly experienced in hereditary haemorrhagic telangiectasia and TTCE, therefore the results may not apply to other centres. Secondly, the level of agreement could be influenced by the presence of only a few patients with a large pulmonary right-to-left shunt. Thirdly, the number of cardiac right-to-left shunts might be underestimated because we only studied the contrast injections at rest focussing on the presence of pulmonary right-to-left shunts. This resulted in a prevalence of 3% compared with 23% in previous studies [30]. Because we used the four-beat rule when the shunt origin could not be visualised, the false positive risk for pulmonary right-to-left shunt is small.

## Conclusion

TTCE has an excellent inter-observer and inter-injection agreement for both the presence and grading of pulmonary right-to-left shunts.

### Take home message

- Moderate to large pulmonary right-to-left shunts are associated with severe neurologic complications in hereditary haemorrhagic telangiectasia.
- Transthoracic contrast echocardiography is recommended for screening of pulmonary right-to-left shunts, although reproducibility has not been described previously.
- This prospective single-centre study showed that transthoracic contrast echocardiography has an excellent inter-injection and inter-observer agreement for both the presence and grade of pulmonary right-to-left shunts.

## Caption Electronic Supplementary Material

Video 1 Pulmonary right-to-left shunt grade 1. Transthoracic contrast echocardiogram, apical 4chamber view.

Video 2 Pulmonary right-to-left shunt grade 2. Transthoracic contrast echocardiogram, apical 4chamber view.

Video 3 Pulmonary right-to-left shunt grade 3. Transthoracic contrast echocardiogram, apical 4chamber view.
